# Comparison of ozonesonde measurements in the upper troposphere and lower Stratosphere in Northern India with reanalysis and chemistry-climate-model data

**DOI:** 10.1038/s41598-023-34330-5

**Published:** 2023-05-02

**Authors:** Suvarna Fadnavis, Archana Sagalgile, Sunil Sonbawne, Bärbel Vogel, Thomas Peter, Frank G. Wienhold, Ruud Dirksen, Peter Oelsner, Manish Naja, Rolf Müller

**Affiliations:** 1 Indian Institute of Tropical Meteorology, Center for Climate Change Research, Pune, India; 2 Forschungszentrum Jülich GmbH, IEK-7, Jülich, Germany; 3 Institute for Atmospheric and Climate Science (IAC), Swiss Federal Institute of Technology (ETH), Zürich, Switzerland; 4 Deutscher Wetterdienst (DWD) GRUAN Lead Centre, Meteorologisches Observatorium Lindenberg, Tauche, Germany; 5 Aryabhatta Research Institute of Observational Sciences (ARIES), Nainital, India

**Keywords:** Environmental sciences, Chemistry

## Abstract

The variability and trend of ozone (O_3_) in the Upper troposphere and Lower Stratosphere (UTLS) over the Asian region needs to be accurately quantified. Ozone in the UTLS radiatively heats this region and cools the upper parts of the stratosphere. This results in an impact on relative humidity, static stability in the UTLS region and tropical tropopause temperature. A major challenge for understanding ozone chemistry in the UTLS is sparse observations and thus the representation of precursor gases in model emission inventories. Here, we evaluate ozonesonde measurements during August 2016 at Nainital, in the Himalayas, against ozone from multiple reanalyses and the ECHAM6-HAMMOZ model. We find that compared to measurements both reanalyses and ECHAM6-HAMMOZ control simulation overestimate ozone mixing ratios in the troposphere (20 ppb) and in the UTLS (55 ppb). We performed sensitivity simulations using the ECHAM6-HAMMOZ model for a 50% reduction in the emission of (1) NOx and (2) VOCs. The model simulations with NO_X_ reduction agree better with the ozonesonde observations in the lower troposphere and in the UTLS. Thus, neither reanalyses nor ECHAM6-HAMMOZ results can reproduce observed O_3_ over the South Asian region. For a better representation of O_3_ in the ECHAM6-HAMMOZ model, NO_X_ emission should be reduced by 50% in the emission inventory. A larger number of observations of ozone and precursor gases over the South Asian region would improve the assessment of ozone chemistry in models.

## Introduction

From the densely polluted South Asian region high amounts of pollutant gases are emitted leading to ozone production in the troposphere^1^. Tropospheric ozone is a major pollutant causing detrimental effects on health, agricultural production, and ecosystems^2,3^. Other than affecting air pollution, ozone in the troposphere and stratosphere is a key constituent of the Earth's radiative balance and atmospheric chemistry^4–10^. The tropospheric ozone concentration over the South Asian region was increasing at a rate of + 0.9% year^−1^ during 1979–2005. Also, GEOS-Chem model simulations show a mean annual trend of 0.19 ± 0.07 (*p* value < 0.01) ppbv yr^−1^ in Indian lower tropospheric ozone during 1990–2010^11^. Surface ozone concentration show a rate of increase of 0.04–0.05 ppb per year during 1990–2013^12^. The rising trends in ozone are due to increasing anthropogenic activities, among which fossil fuel consumption, industrial processes and biomass burning are the major contributors^13–16^. In the troposphere, ozone photochemistry is governed by the oxidation of volatile organic compounds (VOC_S_) and nitrogen oxides (NO_X_). The South Asian region is NO_X_ limited, i.e. increase in NO_X_ concentrations increases ozone and vice-versa^8,9^.

In the upper troposphere and lower stratosphere (UTLS), the photolysis of ozone precursors drives the ozone concentrations along with transport from the stratosphere^17,18,19^. The ozone concentrations in the UTLS are also affected by long-range transport^3,9^. Transport of pollutant gases during pollution events from forest fires, volcanoes, and meteorological patterns e.g. southern Oscillation, Rossby waves, and gravity waves, etc. are some of the processes that affect the ozone levels at remote locations^20–22^. The tropospheric ozone concentrations are also highly influenced by stratosphere-troposphere exchange processes^5,23^. The STE processes are most dominating in the South Asian region during the monsoon season^24^.

Transport caused by large-scale monsoon convection plays an important role in the redistribution of ozone and its precursor gases in the UTLS^1,9,10^. The satellite and aircraft observations show a maximum in ozone precursor gases and a minimum in ozone concentration in the monsoon anticyclone (30°E–130°E, 12°N–45°N)^24–27^. Modeling studies and satellite retrievals suggest that observed maxima of ozone precursor gases are due to the vertical transport of polluted air from the Asian boundary layer to the monsoon anticyclone caused by the large-scale monsoon convection^1,26,28^.

During the monsoon season, heat-driven circulation at the southern slopes of the Himalayas plays an important role in modulating STE processes and hence the ozone concentrations over South and East Asia^1,5^. However, the variability of ozone at the southern slopes of the Himalayas is poorly understood due to the lack of high-resolution in-situ ozone measurements. Satellite datasets are used for this purpose; however, satellite products are of coarse resolution and show biases in comparison with ozonesonde observations. For example, the comparison of Atmospheric Infrared Sounder (AIRS) satellite measurements with ozonesonde data over Beijing shows a 20% high bias in the middle troposphere and a 20% low bias near the stratospheric ozone layer^29^. This study^29^ also indicated a positive correlation and consistent ozone variability between AIRS and ozonesondes profiles in the UTLS regions. Other studies on ozonesonde comparison with satellite observations also show a higher bias (10–45%) in satellite retrievals^30–32^.

The reanalysis and assimilation products e.g. Atmospheric community model (CAMS)^33^, Modern-Era Retrospective analysis for Research and Applications (MERRA2)^34^, and ECMWF Reanalysis v5 (ERA5)^35^ are widely used to investigate the UTLS ozone variability. However, ozone representation in these data sets is subject to the assimilation of in-situ observations and emission inventories. Although a number of satellite data are assimilated in reanalyses (when available, section "Reanalysis data"), the sparse in-situ measurements over South and East Asia limit the accurate representation of ozone in the above data sets, e.g. there are rather few ozonesonde measurements in India and China^31,36^.

Here, we compare the observed profiles of ozonesondes with reanalysis (ERA5, CAMS, and MERRA2), global chemistry-climate model simulations, and ECHAM6-HAMMOZ model results in the UTLS over Asian summer monsoon (ASM) region at Nainital (29.35°N, 79.46°E), India located at the southern slopes of Himalayas. The ozonesonde observations were obtained during the ASM season in August 2016. The study focuses on the evaluation of ozone in the UTLS region, however, a comparison in the troposphere is also provided. We also identify the similarity in ozone in the UTLS by comparing the averaged profile on pressure levels. The characteristics of ozonesonde, chemistry model simulation (ECHAM6-HAMMOZ), and reanalysis (ERA5, CAMS) are provided from probability distribution functions. Thus, in this paper we evaluate ozone profiles during August 2016 obtained from reanalysis (ERA5, CAMS, MERRA2) and the chemistry-climate model ECHAM6-HAMMOZ against ozonesonde measurements at Nainital, in the Himalayas. Our evaluation shows that neither reanalysis nor the chemistry-climate model results could reproduce ozone profiles in the Himalayan region. Hence, we provide options for reducing biases in ozone by emission sensitivity experiments; i.e., reducing the emission of NOx and VOCs by 50% (described in methodology section "Methodology: in-situ, satellite and reanalysis, data sets and chemistry-climate model experiments"). We demonstrate that the reduction of emission of NOx by 50% could reproduce the extreme event of low ozone on 15 August 2016. Using trajectory analysis, we further report that the reason for low ozone on this day is caused by oceanic ozone-poor airmasses uplifted in typhoon Omais.

## Comparison of ozonesondes with reanalysis and ECHAM6-HAMMOZ simulations

Here, we compare measured ozonesonde profiles with reanalysis (ERA5, CAMS, and MERRA2) and ECHAM6-HAMMOZ model (ECHAM-CTL) information (coincide in time). The profiles coincide in time with reanalysis data and the ECHAM-CTL model. These profiles are extracted at a grid centered over Nainital for the time period of the ozonesonde measurements. The mean profile of the ozone mixing ratio from reanalysis and ECHAM-CTL datasets, along with the standard deviation, are discussed to understand the relative differences.

### Mean ozone (O_3_) profiles

**Figure 1:**
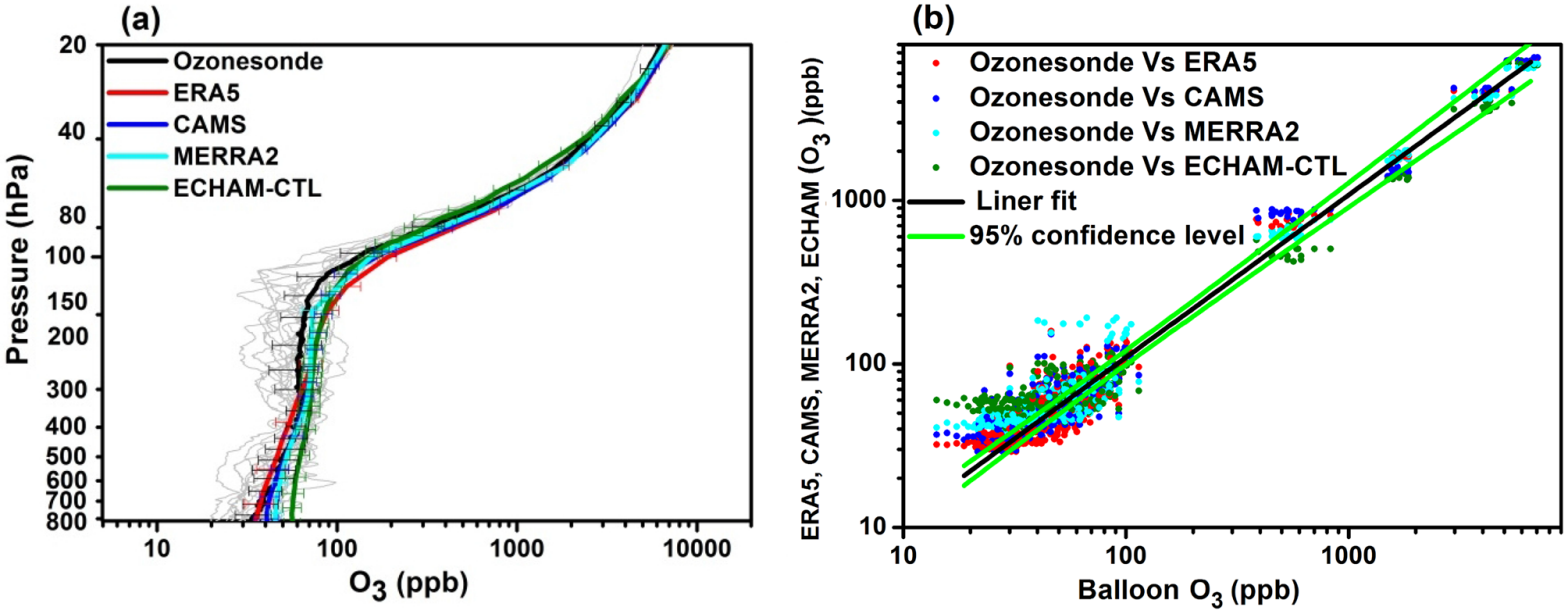
(**a**) ECMWF derived mean ozone profile ERA5 (red), CAMS (blue), MERRA2 (cyan) and ECHAM-CTL (green), and mean ozonesonde profile (black) and each day ozonesondes profile (grey) as a function of pressure with standard deviation, (**b**) Scatter plot of ozonesondes profile vs ERA5 (red), CAMS (blue), MERRA2 (cyan) and ECHAM-CTL (green), respectively for campaign periods. Black lines show the linear fit, and the light green line shows the 95% confidence level of the data sets. (Figure created using the Origin (OriginLab, Northampton, MA)).

Figure 1a shows the measurement of ozone mixing ratio (O_3_) from all ozonesondes (grey) as a function of pressure (from 800 to 20 hPa) along with its mean profile, in comparison with mean profiles of ERA5, MERRA2, CAMS, and ECHAM-CTL. It shows differences in ozone variation with height (pressure) in all the data sets.

In the troposphere, between 800 and 580 hPa, the ozonesonde profile show agreement with ERA5 and the CAMS, while MERRA2 and ECHAM-CTL overestimate than ozonesonde profile by 10 ppb and 18 ppb, respectively (Fig. 1a). At the levels between 580 to 200 hPa, CAMS and MERRA2 shows agreement with ozonesonde data while ERA5 underestimates the observed ozone by 14 ppb and ECHAM-CTL overestimates observed ozone by 55 ppb.

Figure 1a shows that in the UTLS between 200 to 100 hPa, all datasets overestimate ozone by ~ 20 ppb and between 60 to 20 hPa by ~ 200–500 ppb. Between pressure levels 100 to 80 hPa, the ECHAM-CTL simulation shows good agreement with ozonesondes while ERA5, CAMS, and MERRA2 show overestimation by ~ 25 to 150 ppb. Between 80 to 40 hPa pressure levels, ECHAM-CTL shows underestimation by ~ 75 ppb and overestimation by 300 ppb 40 to 20 hPa. ERA5, CAMS, and MERRA2 show overestimation by 300–700 ppb between 80 and 20 hPa. There is a large variation within daily ozonesonde profiles. These temporal variations may be due to synoptic weather systems. During the first half of August 2016 several tropical storms occurred in the western Pacific i.e. typhoon Omais^37,38^. Also, there are differences between the mean profiles of ozonesondes, reanalysis, and ECHAM-CTL. This may be due to various reasons, e.g. spatial resolution; the ozonesondes measurements are at point location while reanalysis and model simulations are at the grid nearest to the station. Also, differences in emission inventory and assimilated data were used in the reanalysis and ECHAM-CTL model processes.

The scatter plot of ozone concentration from ozonesondes versus ERA5, CAMS, MERRA2, and ECHAM-CTL datasets is shown in Fig. 1b. Figure 1b shows that the large numbers of data points for ozone values between 20 and 100 ppb are outside the 95% confidence level. From Fig. 1a, one can see that ozone values 20–100 ppb are found in the troposphere (800–200 hPa). Thus from Fig. 1a, b, we can infer that large variation within the data sets occurs in the troposphere. Similarly, data points for the ozone values 100–1000 ppb are also outside the 95% confidence level (see Fig. 1b). Figure 1a shows that ozone values 100–1000 ppb are found between 200 and 60 hPa. However, data points for ozone values 2000–10,000 ppb (which are between 60 and 20 hPa levels, see Fig. 1a) are mostly within the 95% confidence limits. Thus differences between data sets are less between 60 and 20 hPa levels and within the 95 confidence limits. ^39^also found a similarity in the measurement of ozone from balloon soundings and Aura Microwave Limb Sounder (MLS) in the stratosphere (60–10 hPa) over the Tibetan Plateau.

### Probability Density Function (PDF) analysis

**Figure 2:**
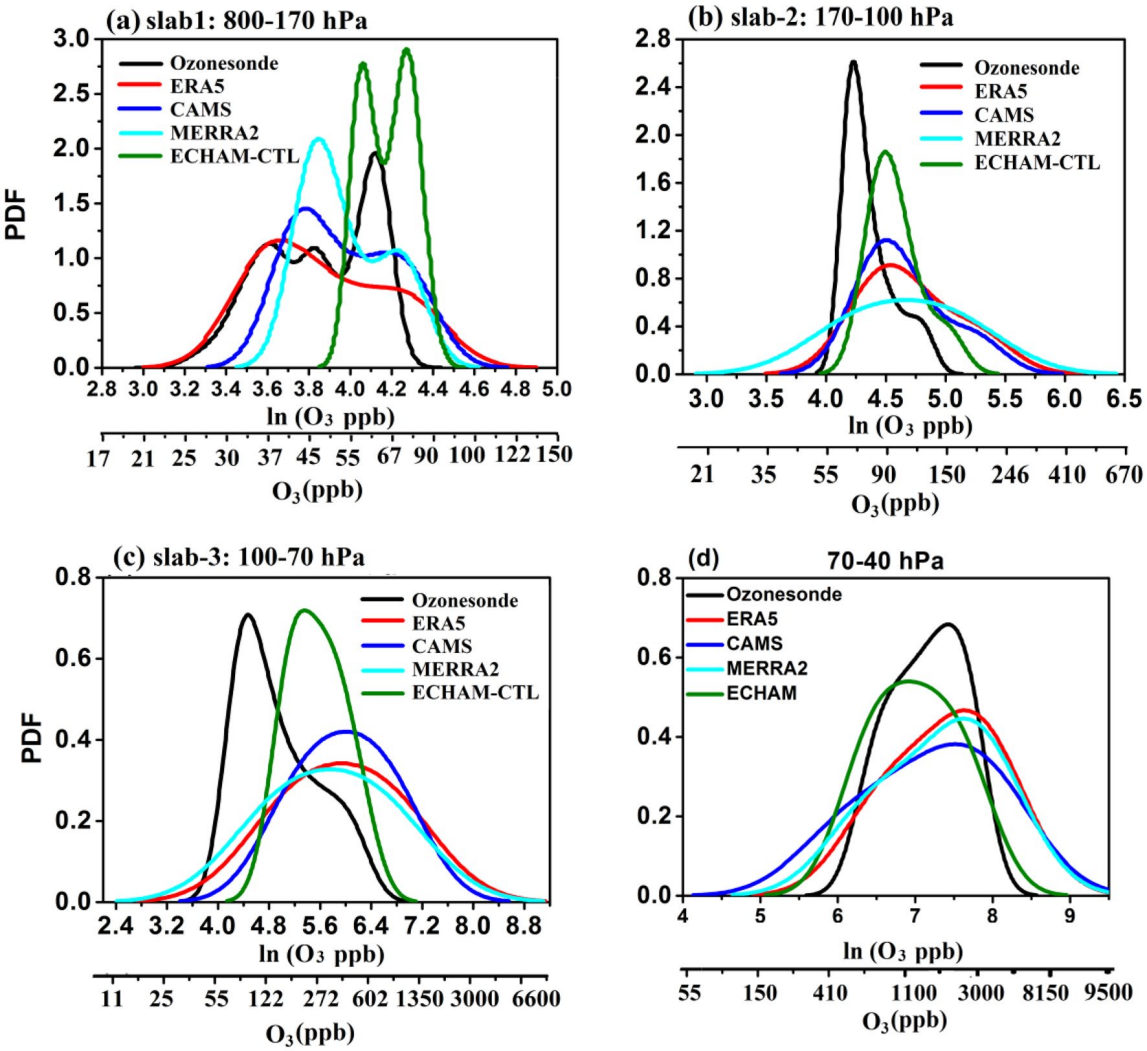
Probability density function (PDF) of ozone mixing ratio (ppb) for ozonesonde measurements at Nainital (black), ERA5 (red), CAMS (blue), MERRA2 (cyan), and ECHAM-CTL (green) for slabs, (**a**) slab-1: 800–170 hPa, (**b**) slab-2: 170–100 hPa, (**c**) slab-3: 100–70 hPa, (**d**) slab4: 70–40 hPa. X-axis is represented in ln (O_3_ in ppb) and also O_3_ in ppb. (Figure created using the Origin (OriginLab, Northampton, MA)).

Figure 2 illustrates the probability distribution functions (PDF) of ozone mixing ratio from ozonesondes, ERA5, CAMS, MERRA2, and ECHAM-CTL for the campaign period at different slabs of atmospheric pressure levels, one in the troposphere (slab-1:800–170 hPa), and three slabs in the UTLS, (slab-2: 170-100 hPa, slab-3:100-70 hPa, Slab-4: 70–40 hPa).

Figure 2a shows the PDF for slab-1. It shows the width (difference between starting and end point of the PDF curve) of the PDF curve is largest in ERA5 (21 to 135 ppb), followed by CAMS (28 to 120 ppb), MERRA2 (30.5 to 100.5 ppb), ozonesondes (19 to 82.5 ppb), then ECHAM-CTL (45.5 to 95 ppb). Thus the width of PDF from ECHAM-CTL is narrower than other data sets. The PDF distribution of reanalysis and ECHAM-CTL shows a bimodal distribution. However, a PDF distribution of ozonesondes measurements shows the normal distribution. Figure 2a depicts ozonesondes PDF curve peaks at ozone concentration 37 ppb, ERA5 at 39 ppb, CAMS at 44 ppb, MERRA2 at 46.6 ppb, and ECHMA-CTL at 58 ppb. All data sets show an overestimation of PDF peak by 2–21 ppb than ozonesonde mean profile in the troposphere region.

In the UTLS, for slab-2 (Fig. 2b) width of ozonesondes PDF curve (50.5 to 224 ppb) and ECHAM-CTL (51.5 to 235 ppb) is smaller than all data sets, CAMS (37 to 399 ppb), ERA5 (32.5 to 478 ppb) and then MERRA2 (18.3 to 628 ppb). For slab-2, all data sets show overestimation (by 22–37 ppb) in comparison to ozonesondes. PDF curve of all data sets shows the normal distribution. In slab 3 (Fig. 2c) width of the MERRA2 PDF curve is largest (11.5 to 7450 ppb) than all other data sets. It is followed by ERA5 (14.5 to 7490 ppb), CAMS (30.8 to 5180 ppb). The width of the PDF curve of ozonesondes and ECHAM-CTL data sets are almost similar (vary between 55.8 to 1100 ppb). The PDF curve peak is overestimated in all re-analysis and model data sets in comparison with the ozonesondes PDF curve (ERA5: 399 ppb, CAMS: 405 ppb, MERRA2: 313 ppb, and ECHAM-CTL: 214 ppb). In the slab-4 (Fig. 2d) the peak of the PDF curve for ERA5, CAMS, and MERRA2 show an overestimation by 200–380 ppb compared to ozonesondes while ECHAM-CTL shows an underestimation by 300 ppb compared to ozonesondes.

**Figure 3:**
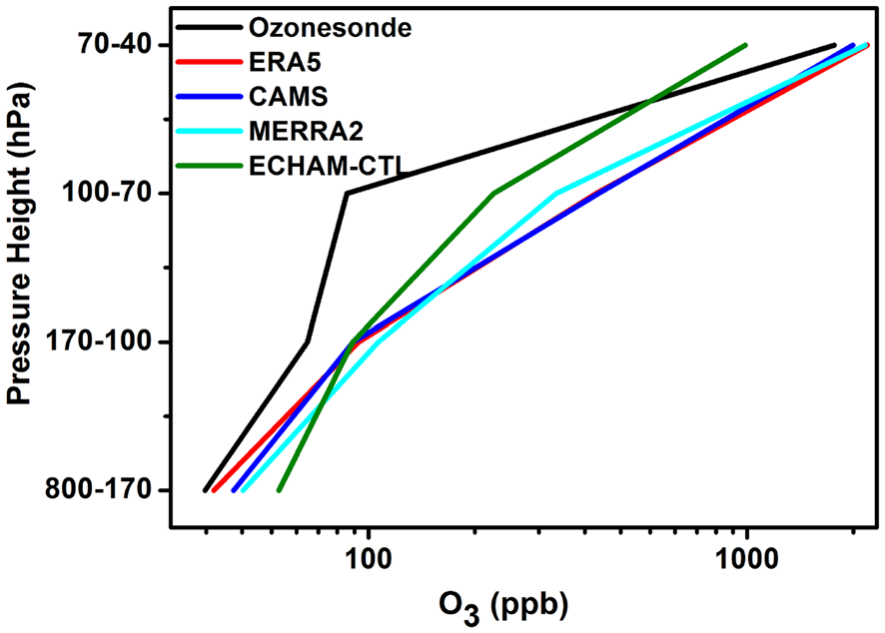
The peak value of ozone mixing ratio from PDF curve at different pressure levels ozonesonde (black), ERA5 (red), CAMS (blue), MERRA2 (cyan), and ECHAM–CTL (green). (Figure created using the Origin (OriginLab, Northampton, MA)).

We plot a peak value of the PDF curve for different pressure slabs in Fig. 3. Figure 3 also suggests that ECHAM-CTL overestimates ozone in the troposphere, between 800 and 100 hPa. A peak in ECHAM-CTL PDF is near the ozonesondes, while it is slightly underestimated above 70 hPa. The ERA5, CAMS, and MERRA2 data sets show an overestimation of the ozone mixing ratio at all pressure levels (by 20 to 400 ppb). The mean vertical profile and PDF analysis suggests that all data sets ERA5, CAMS, MERRA2, and ECHAM-CTL show an overestimation of ozone in the troposphere compared to ozonesondes. In the UTLS, ECHAM-CTL shows underestimation while ERA5, CAMS, and MERRA2 show overestimation compared to ozonesondes. The ozone profiles at Pohang (36.02°N, 129.23°E) in the Korean Peninsula, in comparison with reanalysis products (MERRA2 and CAMS), also show largely overestimation (by 150 ppb) in the troposphere and stratosphere^40^.

### Sensitivity simulations for NO_X_ and VOCs emissions using the ECHAM6-HAMMOZ model

Figures 1a and 2 show that ozone concentrations are overestimated at pressure levels between 800 and 200 hPa in ECHAM-CTL. In South Asia, the chemical production of tropospheric ozone is mainly from NO_X_, and VOCs. However, other ozone precursors also play a role in ozone production^8,9^. Hence we reduce their emissions in the model emission inventory to reduce ozone overestimation in the ECHAM6-HAMMOZ model. We performed two sensitivity experiments for (1) reducing NO_X_ emission by 50% (ECHAM-NO_X_) and (2) reduction of all VOCs by 50% (ECHAM-VOCs). Further, we compare the monthly mean vertical profile of ozone from ECHAM-NO_X_ and ECHAM-VOCs with ozonesondes.

**Figure 4:**
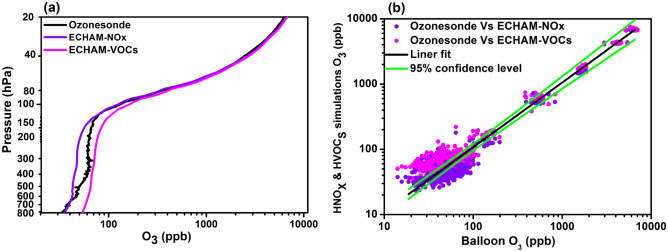
(**a**) Mean profile of ozonesondes (black), ECHAM-NO_X_ (violet), and ECHAM-VOCs (pink) for the campaign period. (**b**) Scatter plot of ozonesonde profile Vs ECHAM-NO_X_ (violet) and ECHAM-VOCs (pink), respectively, for campaign periods. Black lines show the linear fit, and the light green line shows the 95% confidence level of the data sets. (Figure created using the Origin (OriginLab, Northampton, MA)).

In the troposphere, between 800 and 580 hPa, ozonesondes show good agreement with ECHAM-NO_X_ (Fig. 4a). Between 580 and 200 hPa, ECHAM-NO_X_ shows underestimation by 14–15 ppb and ECHAM-VOCs show overestimation by 14–18 ppb. The underestimation in ECHAM-NO_X_ and overestimation in ECHAM-HVOCs simulations at the levels between 580 and 200 hPa may be due to the influence of meteorology/winds in the atmosphere, which is not reproduced in the model. In the UTLS, between 200 and 100 hPa, ECHAM-VOCs simulation shows an overestimation of ozone by ~ 15–60 ppb and between 100 and 40 hPa by ~ 30–285 ppb. Between pressure levels 200 to 120 hPa, the ECHAM-NO_X_ shows underestimation by ~ 8 to 10 ppb. The ECHAM-NO_X_ profile shows good agreement with ozonesondes between 120 and 40 hPa pressure levels. Above 40 hPa, both ECHAM-NO_X_ and ECHAM-VOCs show agreement with each other and ozonesondes. Thus ECHAM-NO_X_ profile shows agreement with ozonesondes between 800 and 580 hPa and 120–20 hPa.

The scatter plot of ozone concentration from ozonesondes versus two sensitivity simulations is shown in Fig. 4b. Figure 4b shows that the large numbers of data points for ozone values between 20 and 200 ppb are outside the 95% significance level. From Fig. 4a, one can see that ozone values of 20–200 ppb are present in the troposphere (800–200 hPa). Thus from Fig. 4a–b, we can infer that large variation within the data sets occurs in the troposphere. Similarly, data points for the ozone values 200–1000 ppb are also outside the 95% confidence level (see Fig. 4b). Figure 4a shows that ozone values 200–1000 ppb are present between 200 and 120 hPa. However, data points for ozone values 2000–10,000 ppb (which are between 120 and 20 hPa levels, see Fig. 4a) are mostly within the 95% confidence limits. Hence differences between data sets are less between 120 and 20 hPa levels and within the 95 confidence limits.

**Figure 5:**
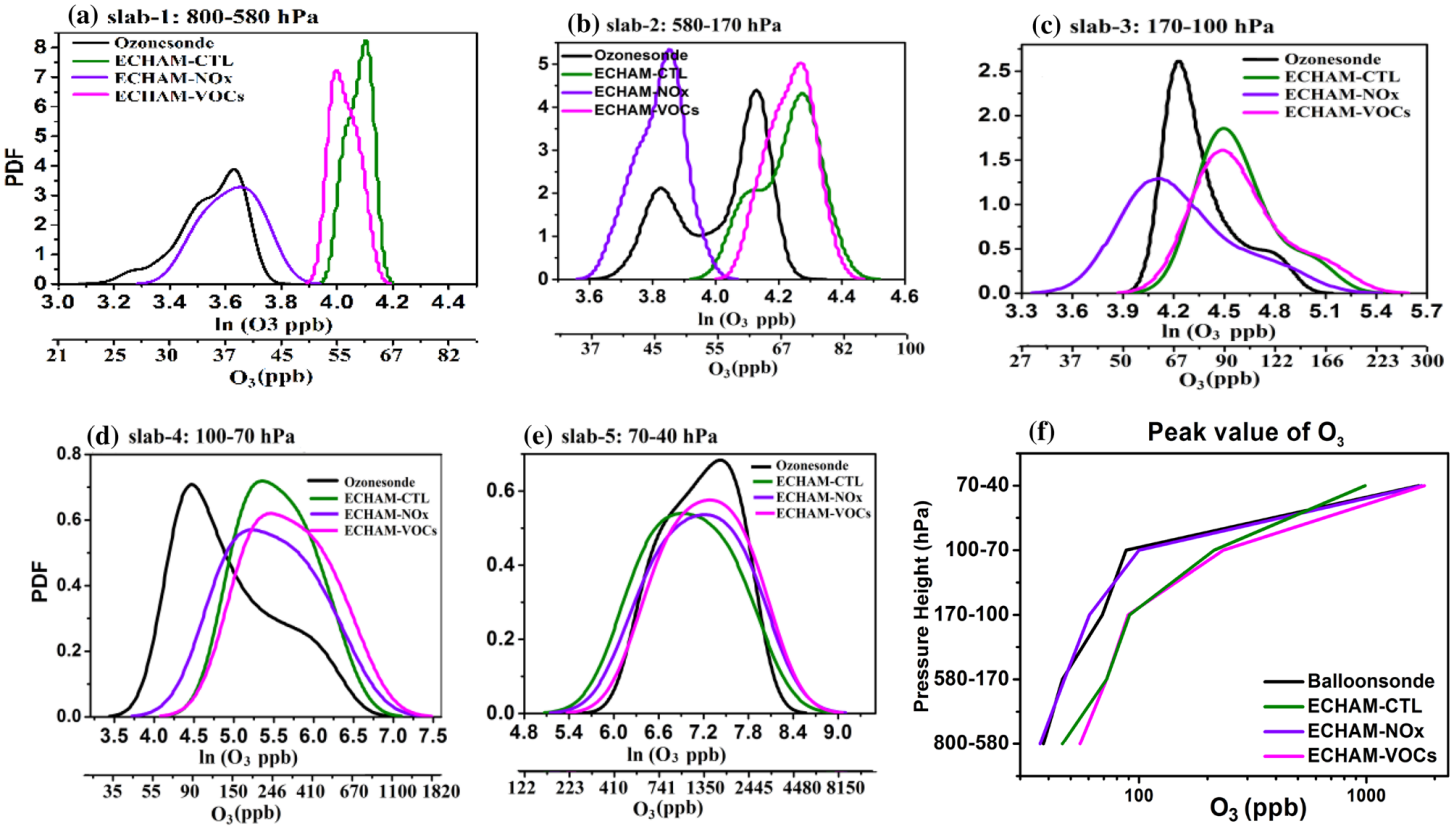
PDF of ozone mixing ratio (ppb) from ozonesonde measurements at Nainital (black), ECHAM-CTL (green), ECHAM-NO_X_ (violet), and ECHAM-VOCs (pink) for the pressure interval, (**a**) slab-1: 800–580 hPa, (**b**) slab-2: 580–170 hPa, (**c**) slab-3: 170–100 hPa, (**d**) slab-4: 100–70 hPa, (**e**) slab-5: 70–40 hPa and (**f**) the peak value of ozone mixing ratio form PDF curve at different pressure levels for ozonesonde, ECHAM-CTL, ECHAM-NO_X_, and ECHAM-VOCs. X-axis is represented in ln (O_3_ in ppb) and also O_3_ in ppb. (Figure created using the Origin (OriginLab, Northampton, MA)).

Figure 5 illustrates the PDF of O_3_ from ozonesondes, ECHAM-CTL, ECHAM-NO_X_, and ECHAM-VOCs for the campaign period at different slabs of atmospheric pressure levels, two in the troposphere (slab-1: 800–580 hPa, and slab-2: 580–170 hPa) and three slabs in the UTLS, slab-3: 170-100 hPa, slab-4: 100–70 hPa, slab-5: 70-40 hPa). Figure 5a shows the PDF for slab-1. The PDF curve of ozonesonde and ECHAM-NO_X_ shows a similar variation and width (36–38 ppb). In contrast, the PDF curve of ECHAM-CTL and ECHAM-VOCs is narrow, containing large values (55–60 ppb). Figure 5a also depicts ozonesondes PDF curve peaks at 38 ppb, which agrees with ECHAM-NO_X_ (at 38 ppb). The ECHAM-CTL PDF peak at 60.5 ppb and ECHAM-VOCs PDF peak at 55 ppb. ECHAM-CTL and ECHAM-VOCs data sets show an overestimation by 17–22 ppb than ozonesondes in the troposphere. But the PDF distribution for ECHAM-NO_X_ for the 800–580 hPa show good agreement with the ozonesondes (Fig. 5a, f), indicating reduction in NO_X_ emissions reduces ozone which improves agreement with observations.

For slab-2 width of PDF curve for the ozonesonde and ECHAM-NO_X_ is similar and smaller (21–51 ppb) followed by ECHAM-VOCs (55–89 ppb), then ECHAM-CTL (50.5–93 ppb). The peak of the PDF curve for ozonesondes at 46 ppb, which agrees with ECHAM-NO_X_ (at 46 ppb), while ECHAM-CTL and ECHAM-VOCs show an overestimation by 26 ppb than ozonesondes. In slab-3 (Fig. 5c) width of the ECHAM-CTL PDF curve (51.5 to 235 ppb), ECHAM-NO_X_ (slab3: 37 to 235 ppb), and ECHAM-VOCs (slab3: 47.5 to 270 ppb) is larger than ozonesondes (slab3: 50.5–224 ppb). In slab-3, the PDF curve peak for ECHAM-NO_X_ shows a small underestimation by 9 ppb and an overestimation in ECHAM-CTL and ECHAM-VOCs by 18–20 ppb than ozonesondes. In slab4 (Fig. 2d) width of the ECHAM-VOCs PDF curve is largest (59.5 to 1820 ppb) than all other data sets, followed by ECHAM-NO_X_ (41.5 to 1540 ppb), ECHAM-CTL (55 to 1100 ppb) and ozonesondes (22.4 to 1000 ppb).

The PDF curve peaks for ECHAM-NO_X_ and shows a small overestimation by 10–50 ppb while ECHAM-CTL and ECHAM-VOCs show a high overestimation by 100–150 ppb than ozonesondes PDF curve (Balloonsonde: 87.7 ppb; ECHAM-CTL: 214 ppb, ECHAM-NO_X_: 138.5 ppb and ECHAM-VOCs: 235 ppb). For slab-5 (Fig. 5e), the peak of the PDF curve for ECHAM-CTL shows an underestimation by 300 ppb, while ECHAM-NO_X_ and ECHAM-VOCs show an overestimation by 5–10 ppb.

The peak value of the PDF curve for different pressure slabs is also shown in Fig. 5f. It is clearly seen that improvement in simulated ozone for ECHAM-NO_X_ sensitivity experiment. It should be noted that the sensitive experiment of ECHAM-NO_X_ shows agreement 800–170 hPa and the UTLS region (70–40 ppb) while it shows underestimation in the troposphere between 170 and 100 hPa regions. The ECHAM-VOCs simulated ozone shows overestimation in all pressure levels.

### Comparison of ozone profiles on a specific day: 15 August 2016

**Figure 6:**
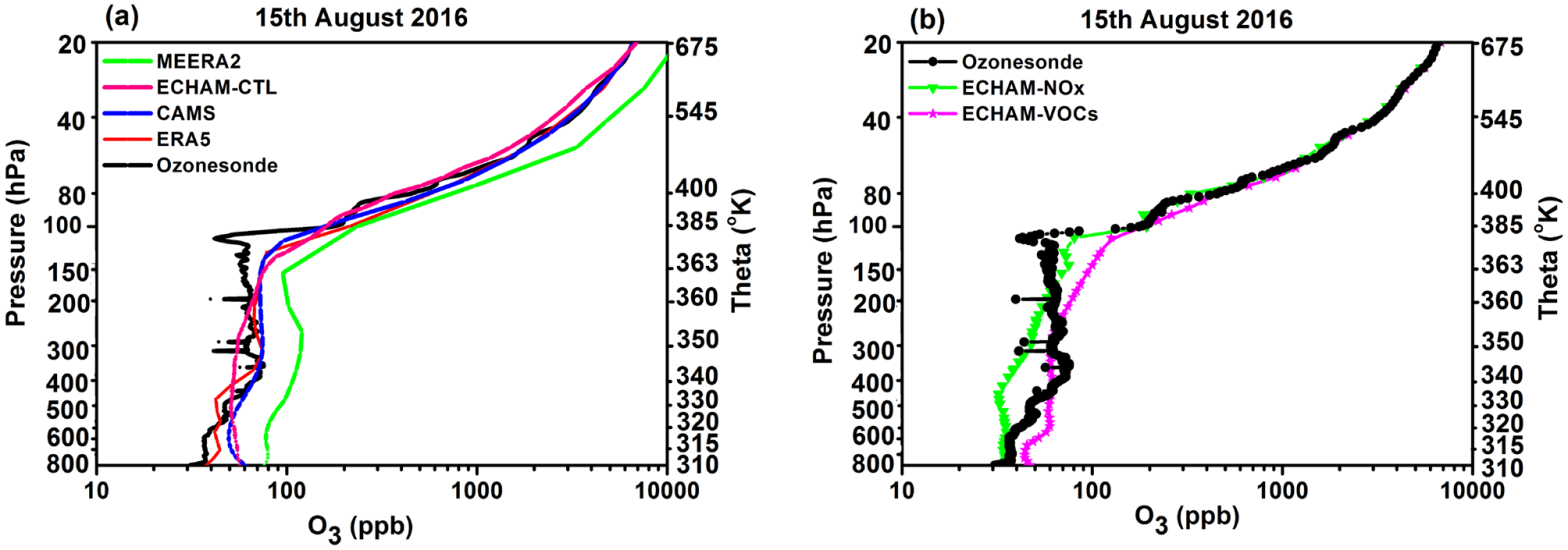
Profiles of ozone on 15 August 2016 at Nainital from ozonesondes (black lines), (**a**) ozonesondes compared to ERA5, CAMS, MERRA2, ECHAM-CTL, (**b**) same as (**a**) but for ozonesondes compared to ECHA-HNO_X_, ECHAM-VOCs. (Figure created using the Origin (OriginLab, Northampton, MA)).

Further, we show a comparison of all data sets on a specific day, the 15th August 2016. For this day, it was shown analysing COBALD measurements (for ATAL detection) of the balloon flight that air masses between 140 and 92 hPa were impacted by typhoon Omais ^37^ which was active between 2 and 12 August 2016 in the western Pacific. However, no ATAL was detected during the balloon flight on 15th August 2016^37^. The ozone profile on this day shows a sharp, deep low ozone concentration (ozone amount decreased by 47 ppb compared to climatology see Fig. S2) near 140 to 100 hPa (363–380 K). This feature of a low ozone amount near 140–90 hPa is seen in all reanalyses and model data (Fig. 6a). On this day, the ERA5 ozone profile shows good agreement with ozonesondes at lower troposphere heights (800–600 hPa). ECHAM-CTL and CAMS also show agreement with the ozonesondes at 600–200 hPa. ECHAM-CTL profile show underestimation compared to ozonesondes between 100 and 20 hPa. ERA5 and CAMS profiles overlap with each other and show good agreement with ozonesondes between 100 and 20 hPa. The MERRA2 ozone profile shows overestimation at all pressure levels.

In Fig. 6b, we compare the measured ozonesonde profile with ECHAM-NO_X_ and ECHAM-VOCs simulations. The ECHAM-NO_X_ profile shows agreement in the lower troposphere, between 800 and 580 hPa, with ozonesondes. A similar agreement is also seen in the mean profile of ECHAM-NO_X_ simulations (Fig. 4a). It is interesting to see that between 100 and 20 hPa, there is a good agreement between ECHAM-NO_X_ and ECHAM-VOCs with each other and with the ozonesondes profile.

**Figure 7:**
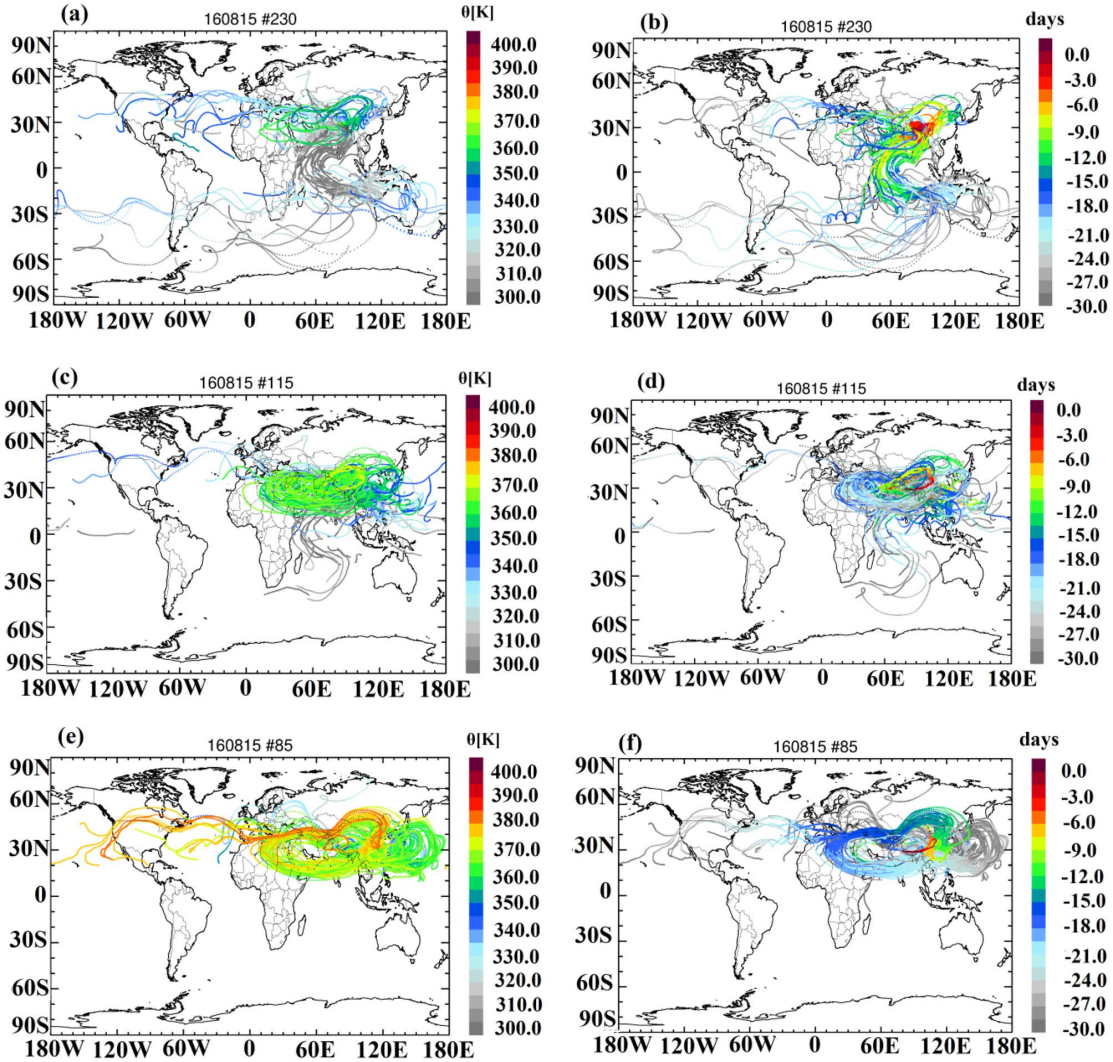
CLaMS 40 d back-trajectories (**a**-**f**), starting at 365–370 K (**a**, **b**), at 370–375 K (**c**, **d**), and at 375–380 K along the balloon profile on 15 August 2016. In panels (**a**), (**c**) and (**e**) the trajectories are colour-coded by potential temperature; in panels (**b**), (**c**) and (**f**), the trajectories are colour-coded by the transport time in days backward in time from the measurements over Nainital. Trajectories were calculated using ERA5 reanalysis^37,41,42^. (Maps generated by using IDL® (https://www.l3harrisgeospatial.com/SofwareTechnology/IDL.aspx)).

Further, we investigate the reason for the low ozone concentration between 140 and 100 hPa (363 to 380 K) on 15th August 2016 using the trajectory module of the three-dimensional Lagrangian chemistry transport model CLaMS (section "Trajectory calculations using the Chemical Lagrangian Model of the Stratosphere (CLaMS)"). Figure 7a–e shows back trajectories initiated at the location of the measurement between 365 and 380 K. Figure 7a–f shows that air masses in the region between 365 and 380 K (140–100 hPa) originate from different regions and different levels of potential temperature. Figure 7e–f shows that air in levels of potential temperature between 375 and 380 K originates mainly in the western Pacific uplifted by typhoon Omais35. Thus clean oceanic air masses (low ozone and low aerosols) are uplifted in the typhoon and subsequently mixed into air within the Asian monsoon anticyclone. Further minor contributions of a stratospheric intrusion transporting ozone-rich stratospheric air along the subtropical jet and subsequent mixing into the anticyclone is indicated by the trajectory calculations. Thus oceanic air reaching the UTLS between 140 and 100 hPa at Nainital is the reason for the observed low ozone on 15th August 2016. This observation is consistent with previous work, where also an impact of air masses uplifted by typhoons on the chemical composition of air within the Asian monsoon anticyclone was identified^37,38,41–44^.

## Discussions and summary

The comparison of ozonesonde profiles with multiple reanalysis (ERA5, CAMS, and MERRA2) and high-resolution chemistry-climate simulations shows that in the troposphere, between 800 and 580 hPa, ozonesonde profile show agreement with ERA5 and the CAMS, while MERRA2 and ECHAM-CTL overestimate the observed ozonesonde profile by 10 ppb and 18 ppb respectively. At the levels between 580 and 200 hPa, CAMS and MERRA2 profiles show agreement with ozonesonde measurements while ERA5 underestimates the measured ozone by 14 ppb and ECHAM-CTL overestimates by 55 ppb compared to the ozonesonde measurements.

A probability density function analysis (PDF) applied to these data sets shows biases in ozone in ERA5 in the troposphere by 3–15 ppb and in the UTLS by 25–400 ppb and CAMS in the troposphere by (8–16 ppb) and UTLS by 20–200 ppb and MERRA2 in the troposphere by 7–11 ppb and UTLS by 37–350 ppb and ECHAM-CTL in the troposphere by 15–21 ppb and UTLS by (80–300 ppb). Thus, our study shows that neither reanalyses (ERA5, CAMS, MERRA2) nor model simulations (ECHAM-CTL) can reproduce measured ozone profiles over the South Asian region during the monsoon season.

Since ozone mixing ratios are overestimated in the ECHAM-CTL simulations, we reduce the emission of (1) Nitrogen oxides (NO_X_) (ECHAM-NO_X_) and (2) all volatile organic compounds (ECHAM-VOCs) (ECHAM-VOCs) by 50% in the model’s emission inventory. These reduced emission model simulations show that ECHAM-NO_X_ simulations show improved agreement with ozonesonde observations in the lower troposphere (between 800 and 580 hPa) and in the UTLS (between 100 and 40 hPa). The ECHAM-NO_X_ and ECHAM-VOCs simulations only slightly underestimate ozone (by 2–7 ppb) between 170 and 100 hPa. The ECHAM-NO_X_ simulation also shows good agreement on 15 August 2016, a special case when low ozone, and no ATAL was observed over Nainital. Our CLaMS trajectory analysis shows that on this day the clean air mass (containing low ozone and low aerosols) from the western Pacific reached 120-100 hPa, which causes the observation of low ozone and possibly of no ATAL on 15th August 2016.

Our study demonstrates that anthropogenic NO_X_ emissions are overestimated in the AEROCOM-ACCMIP-II emission inventory used in the ECHAM-HAMMOZ model simulations over South Asia; they should be reduced by 50% for a better representation of tropospheric ozone in chemistry-climate models. Appropriate simulations of ozone in chemistry-climate model simulations will be helpful for the correct estimation of the oxidising capacity of the troposphere, ozone radiative forcing, ozone heating rates, and the implications for transport processes. Finally, our study suggests that a larger number of height resolved trace gas observations over the South Asian region (including the variability caused by the impact of weather systems such as tropical cyclones) are required for improving the representation of ozone chemistry in models in particular in the Asian monsoon region.

## Methodology: in-situ, satellite and reanalysis, data sets and chemistry-climate model experiments

### Ozonesondes observations

In-situ balloon-borne measurements were performed under the StratoClim project during the Asian summer monsoon at Nainital, Uttarakhand, India (29.35° N, 79.46° E, 1820 AMSL) in August 2016. Nainital is situated at the foothill of the southern slopes of the Himalayas. The Himalayan terrain steeps above 3000 m on the Tibetan Plateau to the north and elevates to the Indo-Gangetic Plain to the south^45^. The measurements were conducted using the payload of the instrument, namely (1) RS41-SGP from VAISALA, Finland, for the measurements of pressure, temperature, and relative humidity, (2) Ozonesonde based on Electrochemical Concentration Cell (ECC) for ozone mixing ratio from EN-SCI, USA^46,47^. These sensors were used with RS41-SGP XDATA interface with the Vaisala DigiCORA MW41 ground receiving sounding system^48^ at a frequency of 1 Hz. The details of measurement technique, resolution, and accuracy can be found in the white paper by Vaisala (WEA-MET-RS-Comparison-White-Paper-B211317En-B) and in Environmental Science (http://www.en-sci.com/). Ozonesondes measures ozone from the ground to 30 km, with a high vertical resolution of ∼100 m. The 1σ uncertainty in the total ozone normalization factor in the tropics is 5.2%^49^. The details of the measurement of campaign sites are elaborated in^45,50^. In this study, we analysed 25 ozonesondes reaching the 20 hPa levels^50^ (see also Table S1).

### Reanalysis data

We compare the ozonesonde measurements with three reanalysis data sets, namely: ERA5, CAMS, and MERRA2. ERA5 is the fifth-generation reanalysis dataset produced by the European Centre for Medium-Range Weather Forecasts (ECWMF), which is the latest global reanalysis dataset. The ERA5 reanalysis is based on the newer IFS cycle 41R2 and provides several improvements compared to ERA-Interim, including higher spatial and temporal resolution^35^. The ERA5 ozone field is the result of the assimilation of the model and satellite observations. All Level-2 ozone products assimilated in ERA5 except METOB-B GOME-2, METEOR-3 and ADEOS-1 TOMS. Since December 2014 the assimilation was switched to the near-real-time product. Additional information on ozone in ERA5 is provided by ozone-sensitive channels of the nadir-viewing infrared sounders (HIRS, AIRS, IASI and CrIS^35,51^. The data set have a temporal resolution of one hour and a spatial resolution of 0.25° × 0.25°, with a vertical range from 1000 to 1 hPa (137 vertical levels).

The CAMS global reanalysis data are produced by the Copernicus Atmosphere Monitoring Service (CAMS)^4^. The CAMS ozone field is the result of the assimilation of satellite observations from GOSAT and METOP-A/B, EOS-Aqua, EOS-Terra, ENVISAT, EOS-Aura, NOAA-14, -16, -17, -18, and -19)^33^, and it integrates from SCIAMARCY, OMI, and GOME/2 as well as ozone profiles from MIPAS and MLS after 2005. The data set have a spatial resolution of 0.75° × 0.75°, with a vertical range from 1000 to 0.1 hPa within 60 hybrid sigma–pressure levels. It gives output every 3 h.

The Modern-Era Retrospective Analysis for Research and Applications, version 2 (MERRA2), is NASA's latest reanalysis, spanning the satellite observing era from 1980 to the present^52^. MERRA-2 assimilates modern hyperspectral radiance and microwave observations, along with GPS-Radio Occultation datasets. It also uses NASA's ozone profile observations that began in late 2004. Additional advances in both the GEOS model and the GSI assimilation system are included in MERRA-2^34^. MERRA2 system produces 3-hourly analyses at 72 sigma-pressure hybrid layers between the surface and 0.01 hPa, with a horizontal resolution of 0.625° × 0.5° with 42 pressure levels (1000 hPa to 1 hPa).

### Chemistry climate model simulations:

We used ozone profiles from the simulation of state of the art ECHAM5-HAMMOZ aerosol-chemistry-climate mode. It comprises the atmospheric general circulation model, ECHAM5^53^, a tropospheric chemistry module MOZ^54^, and an aerosol module Hamburg Aerosol Model (HAM)^55^. The HAM module takes into account the primary aerosol compounds, namely sulphate (SU), Black Carbon (BC), Organic Carbon (OC), sea salt (SS), and mineral dust (DU). The chemistry of ozone, NO_*x*_, VOCs, and other gas-phase species is based on the MOZART-2 chemical scheme based on O_x_-NO_*x*_-hydrocarbons with 63 tracers and 168 reactions^8,9,54^. The anthropogenic and fire emissions are based on the AEROCOM-ACCMIP-II emission inventory. Other details of the model and emissions are reported by^5,28,56,57^.

The model simulations are performed at a T256 spectral resolution, corresponding to 0.5° × 0.5° in the horizontal dimension, while the vertical resolution is described by 31 hybrid σ-p levels from the surface up to 10 hPa (~ 50 km). The simulations have been carried out with a time step of 20 min. Monthly varying Atmospheric Model Intercomparison Project (AMIP) sea surface temperature (SST) and sea ice cover (SIC)^58^ were used as lower boundary conditions. We performed three sets of six member’s ensemble simulations for the period 1 January 2015 to 31 August 2016. The analysis is performed for August 2016 leaving other period as spin-up. The experiment (1) control (referred to as ECHAM-CTL) compare with ozonesondes and reanalysis data. The ECHAM-CTL simulated ozone is overestimated than ozonesonde measurements hence we performed two additional simulations: (2) for a 50% reduction in anthropogenic emissions of Nitric oxides (NO_X_) referred to as ECHAM-NO_X_ (3) for a 50% reduction in anthropogenic emissions of all species of Volatile Organic Compounds (VOCs) referred as ECHAM-VOCs. The spread between six members in 3 sets of experiments is shown in Fig. S1. The advantage of using a high-resolution chemistry-climate model against regional models is for better performance for large-scale monsoon dynamical processes and ASAM^1,9,24,28,57,59^.

### Trajectory calculations using the chemical Lagrangian model of the Stratosphere (CLaMS)

Global Chemical Lagrangian Model of the Stratosphere (CLaMS) simulations^60^ are generally driven by meteorological reanalyses. Here we employ the trajectory module of CLaMS; trajectories are calculated backward in time and are truncated when they reach the model boundary^37,38^. At the beginning of the (backward) trajectory calculation, each air parcel is located at the location of the measurement in August 2016. We use horizontal winds from ERA5 reanalysis^35^ provided by the European Centre for Medium-Range Weather Forecasts (ECMWF). For the vertical velocities, the diabatic approach was applied using the diabatic heating rate as the vertical velocity, including latent heat release^37^.

The aim is to analyse the transport pathways of air masses from the lower troposphere (i.e. from the model boundary) into the anticyclone region and the transport of stratospheric air masses around the Asian monsoon anticyclone to the location of the balloon measurements over Nainital. The (backward) trajectories allow the origin of air masses and their transport pathways to be identified^37,38,41^.

## Supplementary Information

Supplementary Information.

## Data Availability

The data used can be obtained from corresponding author on request.
