# Supplementary material for: Comparison of ozonesonde measurements in the upper troposphere and lower Stratosphere in Northern India with reanalysis and chemistry-climate-model data
Source: Sci Rep. 2023 May 2;13:7133. doi: 10.1038/s41598-023-34330-5 (PMC10154380; doi:10.1038/s41598-023-34330-5)
Supplement: Supplementary file 1 — Supplementary Information. [file 41598_2023_34330_MOESM1_ESM.docx]

**Supplementary Tables and Figures:**

**Table S1**. Number of balloon soundings performed for RS41 and along with ECC Ozone

payload during campaign period. The number of early burst soundings given in

parentheses. Early burst is defined as a burst altitude < 25 km.

| **Campaign** | **Observation Period** | **RS41** | **ECC Ozone** | **Early Burst** |
| --- | --- | --- | --- | --- |
| Nainital, India | 2-31 Aug 2016 | 30 | 28(2) | 2 < 25 Km |


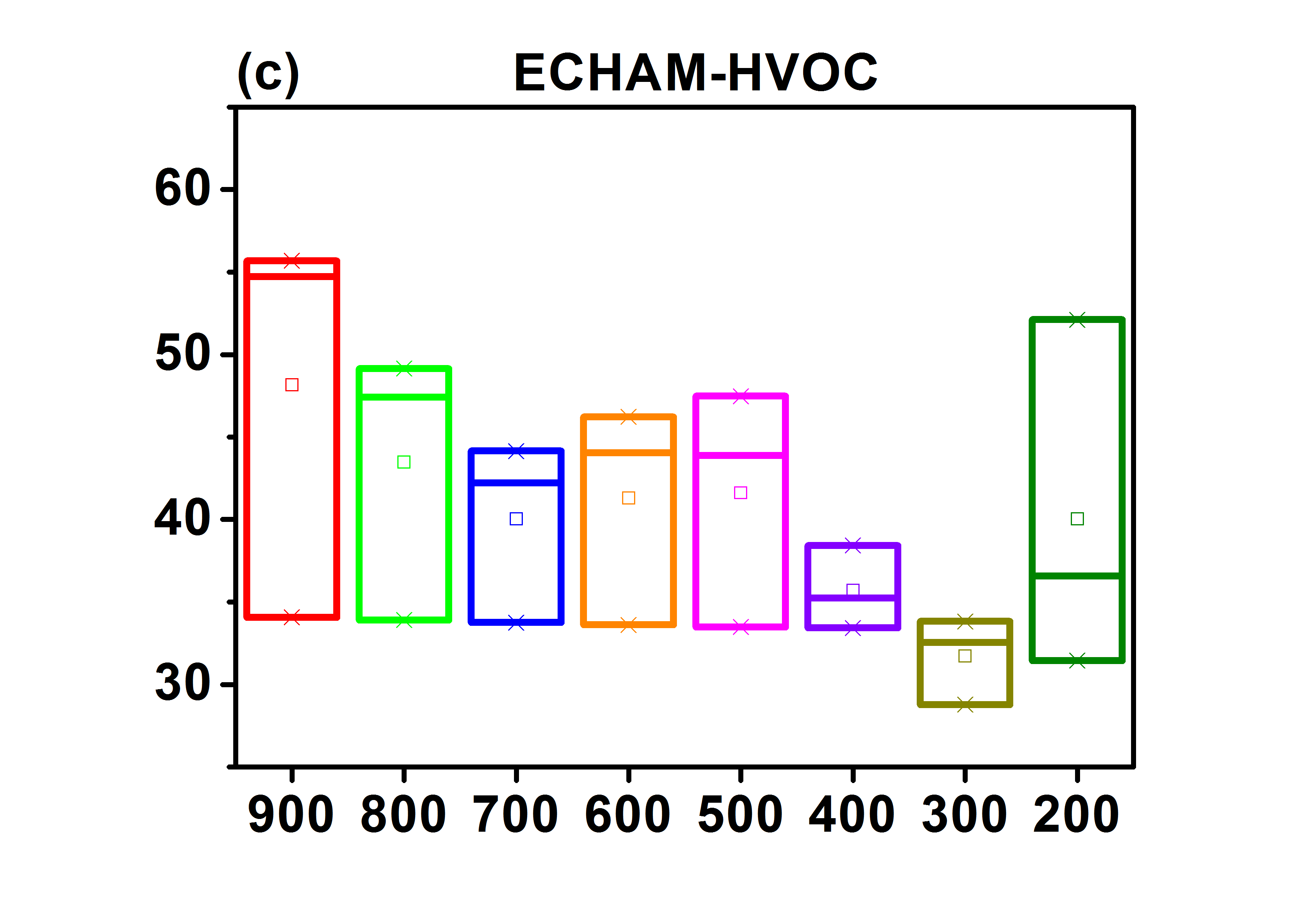

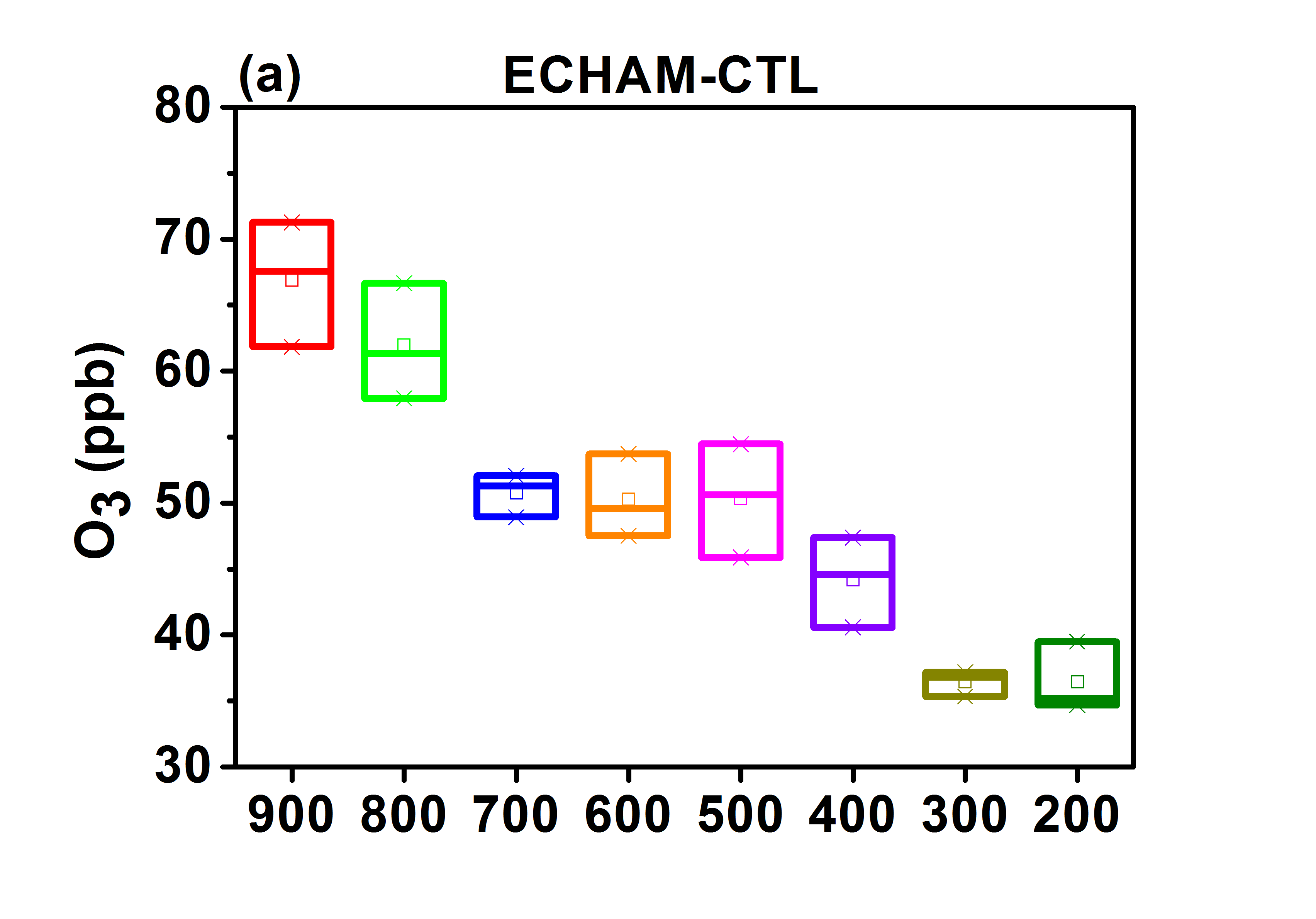

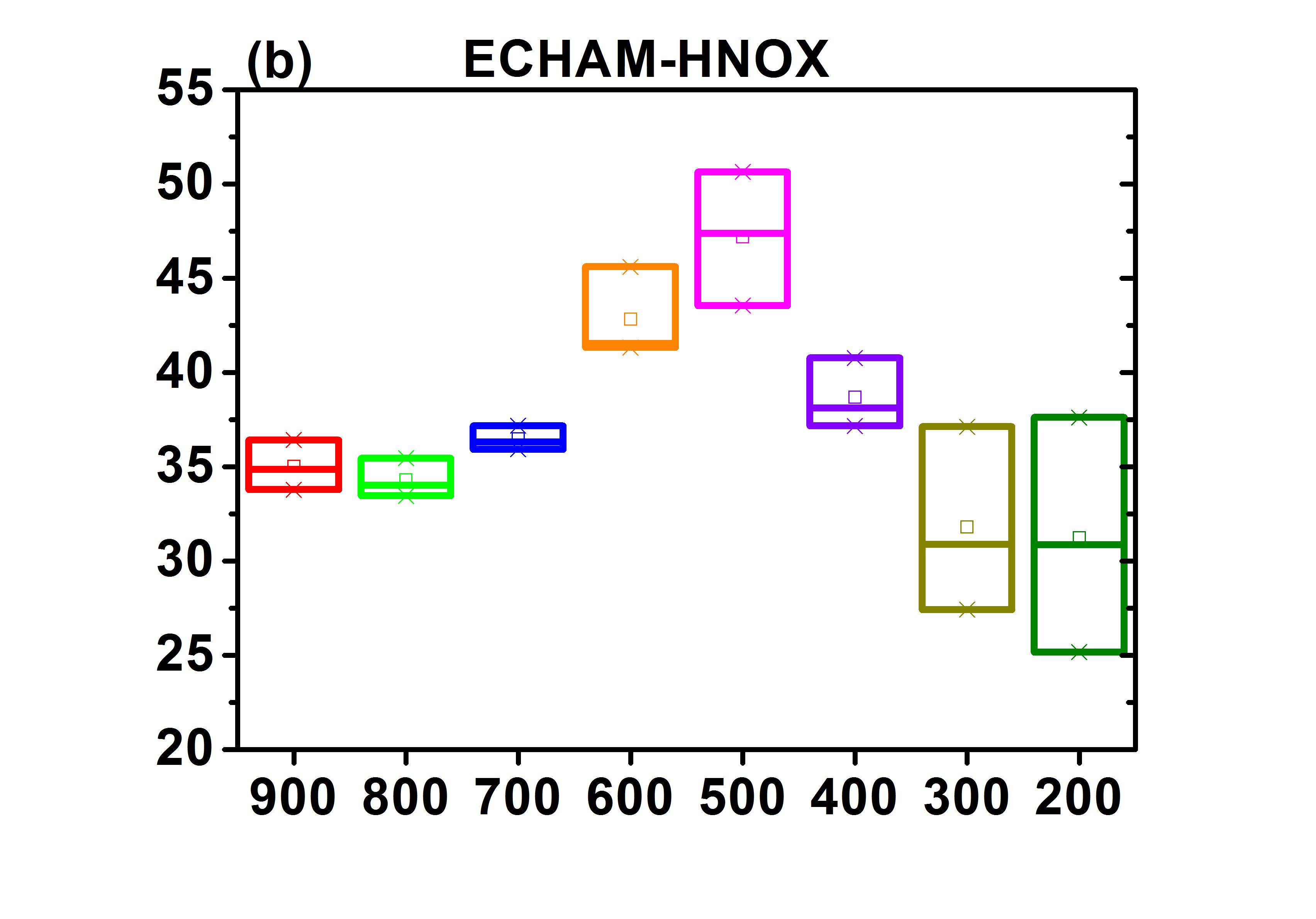

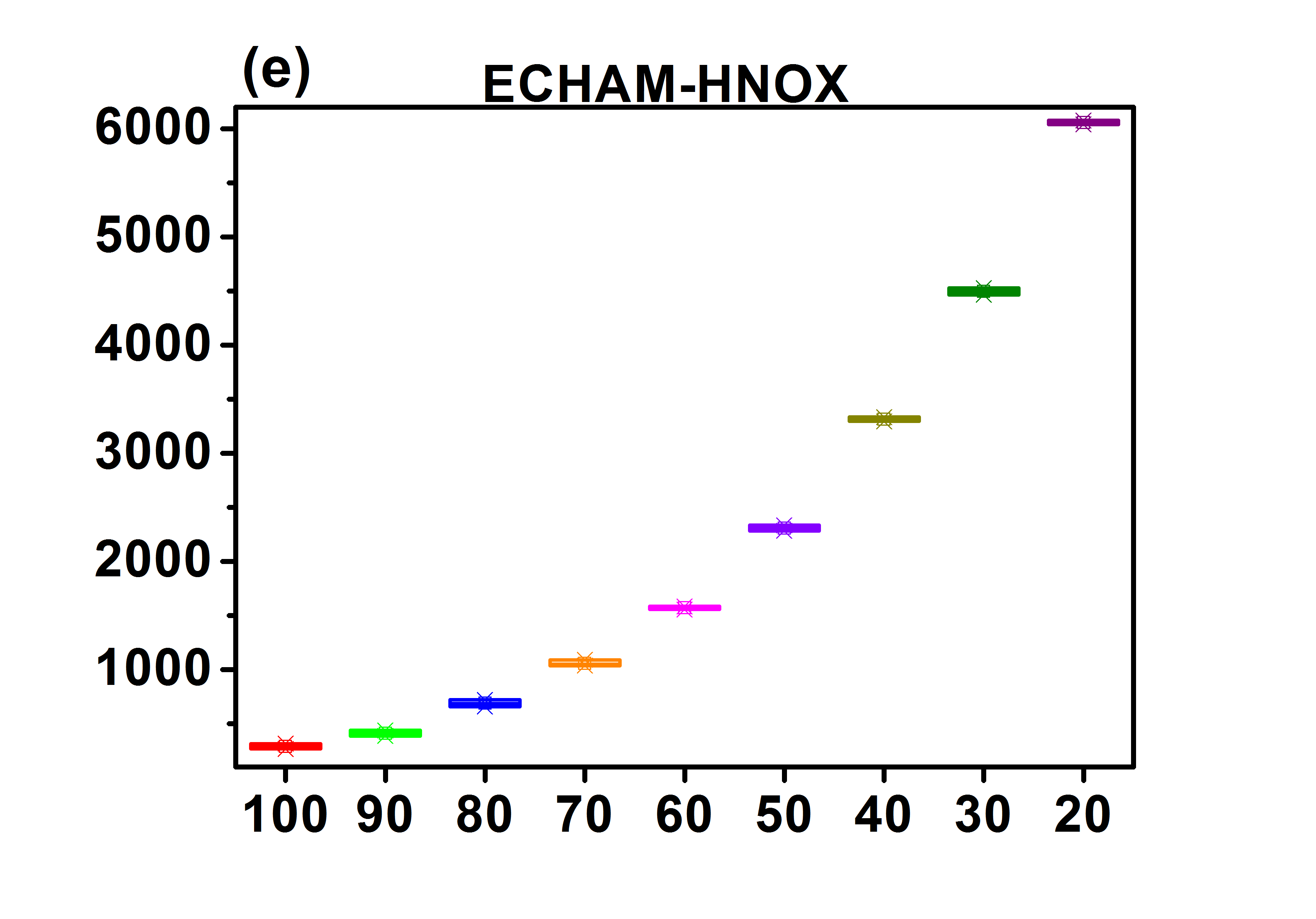

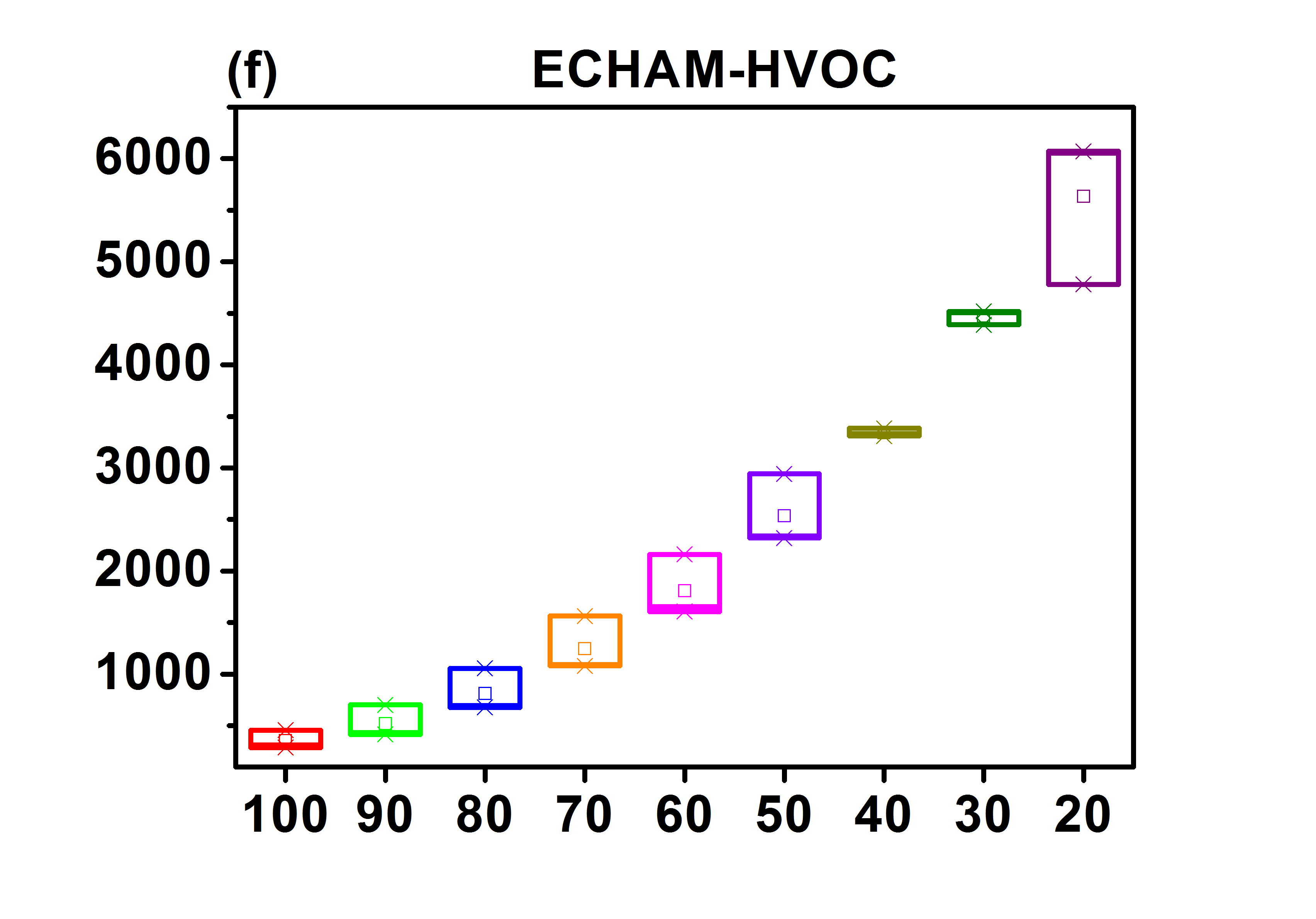

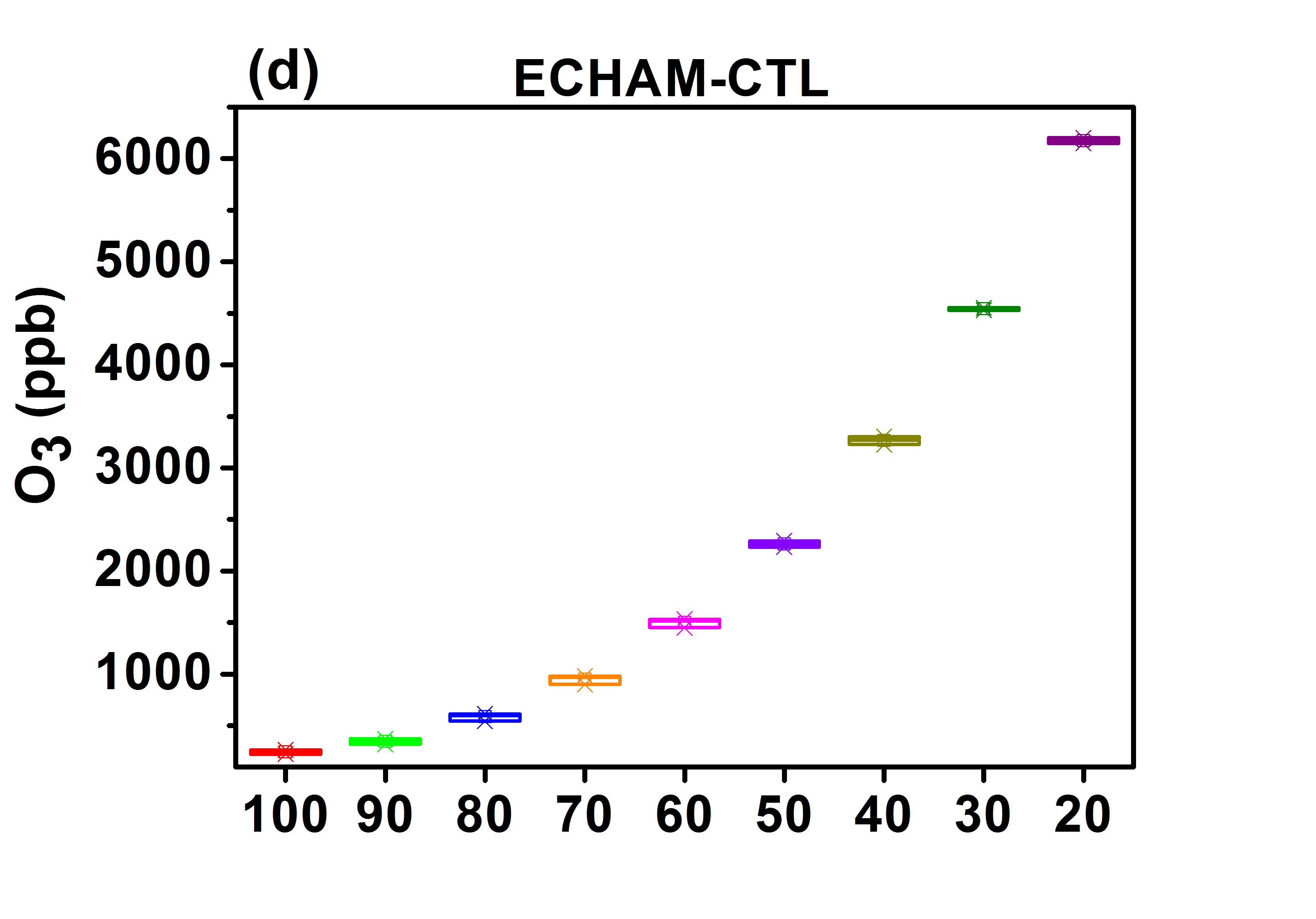


**Figure S1:** Spread between six members of simulations for pressure levels 1000-100 hPa for experiments (a) ECHAM-CTL, (b) ECHAM-NO_X_, and (c) ECHAM-VOCs, (d-f) same as (a-c) but pressure levels 100-20 hPa. (Figure created using the Origin (OriginLab, Northampton, MA)).


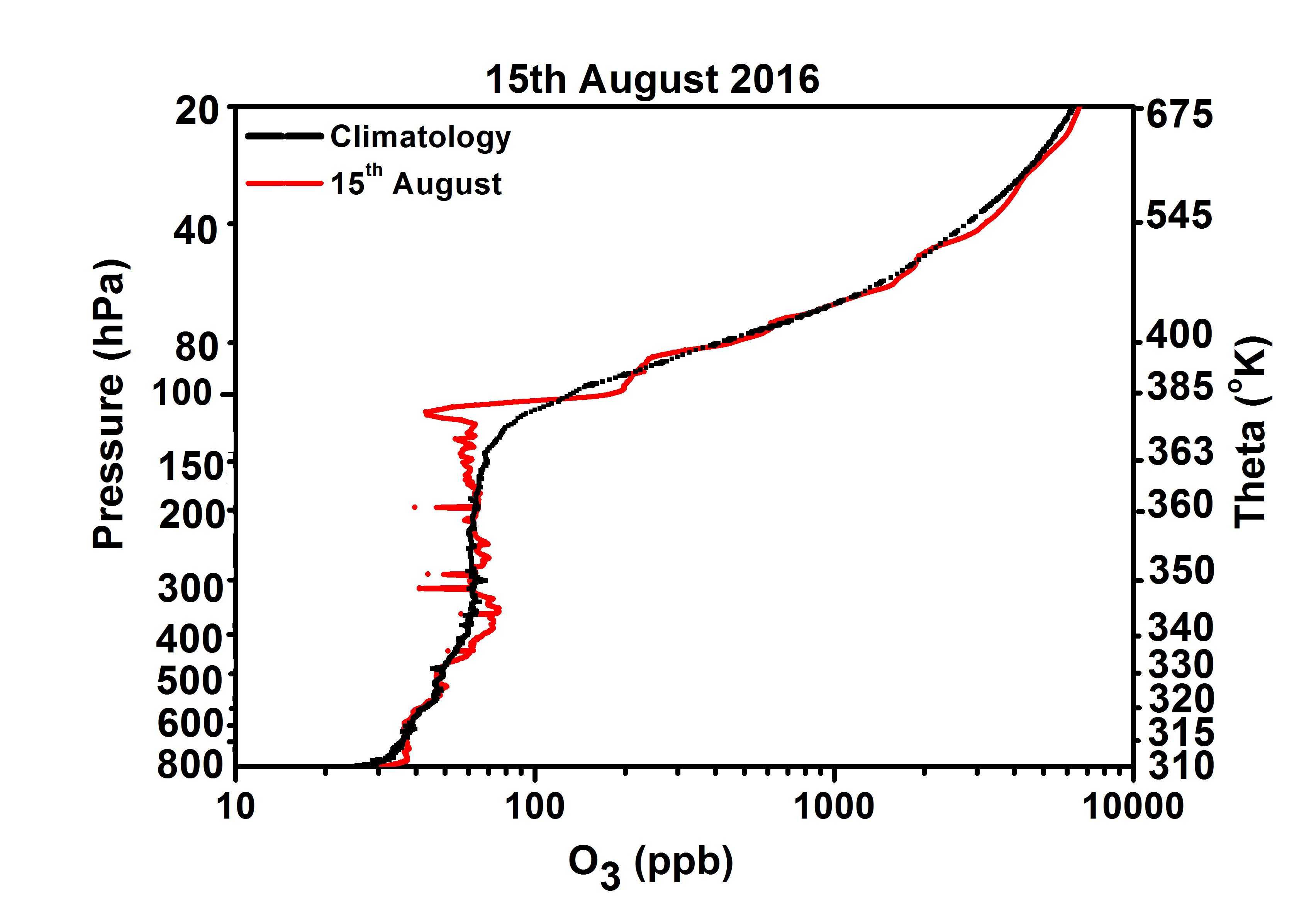


Figure S2: Profiles of ozone on 15 August 2016 at Nainital from ozonesondes (red lines) and climatology (black line). (Figure created using the Origin (OriginLab, Northampton, MA)).
